# Prevalence and associated factors of congenital anomalies in Ethiopia: A systematic review and meta-analysis

**DOI:** 10.1371/journal.pone.0302393

**Published:** 2024-04-30

**Authors:** Wubshet Nebiyu Mogess, Tefera Belsty Mihretie

**Affiliations:** Department of Human Anatomy, School of Medicine, College of Health and Medical Sciences, Haramaya University, Harar, Oromia, Ethiopia; University of the Witwatersrand, SOUTH AFRICA

## Abstract

**Background:**

Congenital anomalies represent a significant contributor to infant mortality, morbidity, and enduring disability. With this in mind, the present investigation endeavour to ascertain the pooled prevalence of congenital anomalies and associated determinants among neonates in Ethiopia.

**Method:**

PubMed, Google Scholar, CINAHAL, Hinari, and Global Health databases were systematically searched. Joanna Briggs Institute (JBI) assessment checklist was used to assess quality of included studies. Data were extracted from database and exported to stataMP-17 for analysis. Pooled prevalence was determined using DerSimonian-Laird random effects model. The degree of heterogeneity and Publication bias were assessed using I^2^ statistics and Eggers test, respectively. Study protocol was registered under PROSPERO ID CRD42021229140.

**Result:**

A total of 18 studies with 519,327 participants were included in the study. Pooled prevalence of congenital anomalies in Ethiopia was 2% (95% CI: 0.02, 0.03%). Among affected newborns neural tube defect (48%) was the most common congenital anomaly in Ethiopia, followed by orofacial cleft (19%). Risk factors such as alcohol consumption (pooled OR: 2.28, 95% CI: 1.54, 3.38), lack of folic acid supplement (pooled OR: 2.83, 95% CI: 1.09–7.36), medication during pregnancy (pooled OR: 2.58, 95% CI: 1.03–6.47), khat (*Catha edulis*) chewing (pooled OR: 2.44, 95% CI: 1.61–3.71), exposure to pesticides (pooled OR: 4.45, 95% CI: 2.44–8.09) and maternal illness (pooled OR:1.79, 95% CI: 1.03, 3.10) had statistically significant association with congenital anomalie**s** in Ethiopia.

**Conclusion:**

In this review, prevalence of congenital anomalies in Ethiopia was high with considerable regional variation. The most common type of congenital anomaly in Ethiopia was neural tube defects, followed by oro-facial cleft. Alcohol consumption, inadequate intake of folic acid, khat chewing, maternal diseases, exposure to pesticides, and use of medication during pregnancy were identified as potential contributors to congenital abnormalities in Ethiopia.

## Introduction

Congenital malformations are structural, functional, or metabolic disorders of prenatal origin, at birth, or throughout life [1,2]. Birth defects are the main cause of morbidity, long-term disability, and death in infants and children [3]. Globally, an estimated 7.9 million infants (6%) are born with birth defects each year [4]. Worldwide, Congenital anomalies cause about 3.3 million deaths annually of this, 94% death is from middle and low income countries [5]. The prevalence of congenital anomalies varies from country to country; it is reported that 4.54% in Eastern Africa [6] and 20.40% In Sub-Saharan Africa [7]. The occurrence of congenital anomalies in different African countries widely varied in Kenya 9.62%, Egypt 7.4%, Uganda 6.62%, Nigeria 2.66% and 1.66% south Africa [5].

The pattern and cause of congenital anomalies may vary depending on geographic location, known complex genetic interactions, environmental factors, and maternal health/nutrition status [8]. Middle and low-income countries are more likely affected by congenital anomalies due to many factors. These include an increased frequency of intrauterine infections, poor maternal nutrition, low socioeconomic and educational levels, lack of environmental protection measures, and poorly regulated access to medicines [9].

Parental location is one of the causes of certain birth defects due to exposure of the pregnancy to toxic environmental factors during embryogenesis [10]. A balanced diet and periconceptional intake of vitamins, especially folic acid and vitamin B12, by the mother and father, can reduce the risk of congenital malformations [11,12].

Neuronal abnormalities and neural tube defects were the most prevalent congenital abnormalities in sub-Saharan Africa [7].

There is a shortage of reports or accurate medical records of congenital anomalies in developing countries [13].

Knowledge of congenital anomalies is useful in identifying evidence of risk factors in situations and in assisting health services in planning and assessing antenatal screening in high-risk populations. In addition, there is a need to raise awareness of birth defects and the use of folic acid/multivitamins or nutritional status to reduce the incidence of birth defects. Therefore, the purpose of this systematic review and meta-analysis was to investigate the pooled prevalence and associated factors affecting congenital anomalies in Ethiopia.

### Review question

The review question was designed by considering CoCoPop (condition, context and population) as follow**s** “what are the prevalence and associated factors affecting congenital anomalies among newborns, neonates, infants and children in Ethiopia”.

## Materials and methods

### Protocol and registration

This review followed PRISMA guideline items and was registered in the International Prospective Register of Systematic Reviews (PROSPERO) 2021 CRD42021229140 Available from: https://www.crd.york.ac.uk/prospero/display_record.php?ID=CRD42021229140.

The methodology of this systematic review and meta-analysis was prepared in accordance with 2009 checklist of the Preferred Reporting Items for Systematic Reviews and Meta-Analyses (PRISMA) [14].

### Eligibility criteria

Studies which report prevalence and associated factors of congenital anomalies and are written in English were included in this review. All of these study methods were observational (case-control, cohort, or cross-sectional) and were conducted in Ethiopia to determine the prevalence and contributing causes of birth abnormalities. The analysis omitted articles for which it was difficult to find outcome data, due to outdated studies published before 2015, articles without full abstracts or texts, and unpublished and peer-reviewed articles.

### Sources of studies and search strategies

Electronic databases such as PubMed, Google Scholar, CINAHAL, Global Health, and Hinari were used to access journals and articles published up to August 2023. The search strategy was used to find all relevant studies in the search database using major Boolean operators. The key searching terms were designed by considering condition, context and population as follows by initial keywords (“Prevalence” OR “Epidemiology” AND “Congenital Anomalies “OR “birth defect” OR “Congenital Malformation” OR “Congenital Disorder” AND “Associated Factors” AND “Ethiopia”).

### Quality assessment and data extraction

The Joanna Briggs Institute (JBI) standardi**s**ed critical appraisal checklist [15] was used to assess the quality of individual studies. This checklist use**s** eight questions for correctional studies and ten for case**-**control studies. The purpose of this critical evaluation was to assess the reliability of the investigations and analyses. The quality assessment checklist was discussed in detail among the investigators. Each article was assessed independently by two authors and any disagreement**s** were resolved through discussion and consensus. Finally, 18 papers matched the criteria for systematic review and meta-analysis as outlined by the Joanna Briggs Institute (Table 1).

**Table 1:** Detailed Description of the studies included to analyze the prevalence and contributing factors of congenital anomalies in Ethiopia: Systematic and meta-analysis.

Author	Year of Publication	Study area	Case	Sample size	Study designs	Prevalence	Associated factors	AOR (95% Cl)	Quality score (8) and (10)
Taye M et al[16]	2019	Amhara	15188	76201	Cross-sectional	1.99%	Alcohol drinking	2.394(1.212,4.726)	6
Seyoum G, Adane F[17]	2018	Amhara	317	19650	Cross-sectional	1.61%	Alcohol drinking	2.02(1.13, 3.6)	7
							Lack of use of folic acid	6.54(3.54, 12.08)	
							Maternal illness	4.05(2.27, 7.21)	
							Medication during pregnancy	3.55(1.98, 6.38)	
Getachew B, et al[18]	2023	Oromia	31	754	Cross-sectional	4.1%	Alcohol drinking	1.282(0.514, 3.195)	7
							Maternal illness	4.3(1.6, 11.37)	
							Chat chewing	3.41(1.46, 7.95)	
							Exposed to pesticides during pregnancy	12.9 (0.91, 32)	
							Maternal age >35	1.06(0.36, 3.16)	
Birhanu K et al[19]	2021	Oromia	78	422	Cross-sectional	18.5%	Lack of use of folic acid	2.4(1.1, 5.02)	5
Mekonen HK etal [20]	2021	Tigray	383	12225	Cross-sectional	2.11%	Maternal illness	1.13(0.775, 1.64)	8
							Lack of ANC	0.909(0.714, 1.157)	
							Birth weight < 2.5 kg	0.849(0.69, 1.046)	
							Medication during pregnancy	0.948(0.75, 1.198)	
							Maternal age >35	2.905(2.059, 4.098)	
Gedamu S et al. [21]	2021	Oromia	23	2218	Cross-sectional	1%	Maternal illness	2.2(0.54, 8.8)	6
							Lack of ANC	3.2(0.8, 12.8)	
							Birth weight < 2.5 kg	0.3(0.1,0.9)	
							Urban residence	0.3(0.1,1)	
							Maternal age >35	6.5(2.4,18)	
Mekonnen D etal[22]	2021	Amhara	69	11177	Cross–sectional	0.62%	Lack of ANC	0.24(0.14, 0.39)	7
							Birth weight < 2.5 kg	4.56(2.75, 7.55)	
							Urban residence	2.16(1.33, 3.51)	
							Maternal age >35	0.53(0.22, 1.26)	
Mekonen HK. et al. [23]	2015	Tigray	32	1516	Cohort study	2%	Lack of ANC	2.13(-4.127,0.124)	6
Geneti SA et al. [24]	2021	Oromia	21	45951	Cross-sectional	5.5%	-		5
Abdu H, Seyoum G.[25]	2019	Amhara	324	22624	Cross-sectional	1.43%	-		6
Silesh M.etal.[26]	2021	Oromia	199	3346	Cross-sectional	5.95%	-		6
Taye M.etal. [27]	2016	Amhara	6076	319776	Cross-sectional	1.9%	-		5
Taye M.etal. [28]	2018	Amhara	207	414	Case-Control	_	Maternal alcohol drinking	2.39(1.87–11.3)	7
Tsehay B. et al. [29]	2019	Amhara	100	398	Case-Control	_	Maternal alcohol drinking	12.7(3.3–48.7)	8
							Lack of use of folic acid	7.3(2.9–18.8)	
							Maternal illness	3.4(1.3–11.6)	
							Living in urban	6.4 (1.9–21.7)	
							Maternal age >35	4.9(1.1–23.68)	
Jemal S.etal.[30]	2021	Oromia	105	418	Case-Control	_	Maternal alcohol drinking	48(1.38–8.74)	6
							Maternal illness	6.1(2.39–15.57)	
							khat chewing	4(1.49–10.65)	
							Medication during pregnancy	0.43(0.16–1.2)	
							Lack of folic acid use	3.25(1.6–6.61)	
Mekonnen AG. etal.[31]	2020	Oromia	136	409	Case-Control	_	Maternal alcohol drinking	2.59(0.93–7.25)	7
							Living in urban	0.56(0.43–5.83)	
Getachew H, Derbew M. [32]	2020	Addis Ababa	200	692	Case-Control	_	Lack of ANC	2.3 (1.5–3.6)	6
							Lack of folic acid use	1.8 (1.1–2.8)	
							Birth weight<2.5 kg	2.3(1.4–3.9)	
Abebe S, et al[33]	2021	Oromia	251	1138	Case-Control	_	Alcohol drinking	1.12(0.508–2.446)	7
							Lack of use of folic acid	0.428(0.247–0.74)	
							Maternal illness	1.18(0.77–1.62)	
							Birth weight<2.5 kg	0.3(0.1-_0.9)	
							khat chewing	1.556(0.797–3.04)	
							Medication during pregnancy	1.303(0.808–2.1)	

AOR, Adjacent odds ratio; CI, Confidence interval; ANC, Antenatal care; kg, kilogram; Sign of (-), Used to indicate absence of prevalence and associated factor.

The ENDNOTE citation manager was used to extract data from various sources and remove duplicate articles. The two data extractors were used during the different review phases, and any discrepancies were resolved through discussion and consensus. Stud**y** characteristics such as author name, publication year, study area, study design, sample size, prevalence, case and associated factors with adjusted risk ratios (OR) were extracted and saved in Microsoft Excel (Table 1) by two authors (T.B and W.N).

### Outcome measures

The main outcome measure were the prevalence of congenital anomalies, which was estimated as the overall number of congenital anomalies divided by the sample size and multiplied by 100. The second outcome was identifying associated factors with a congenital anomaly, which were determined using the odds ratio. The main factors considered in this research were maternal alcohol consumption, maternal illnesses, urban living, and exposure to chemicals during pregnancy, inadequate ANC, and khat (*Catha edulis*) chewing (green leaves that chewed by the people for its stimulant action).

### Data analysis

The extracted data were exported to STATA **v**ersion 17 for analysis of pooled estimates of prevalence and associated factors of congenital anomalies in Ethiopia. The **s**tatistical heterogeneity and publication bias of the studies were examined using forest plots and I^2^ heterogeneity tests. Qualitatively examine publication bias with a visual inspection of the funnel plot diagram. Additionally, I^2^ values of 25%, 50%, and 75% were taken to represent low, medium, and high heterogeneity respectively [34,35]. To view the cause of heterogeneity meta- regression analysis was performed. The results were presented using text, tables and forest diagrams with effect measures and a 95% confidence interval.

## Result

### Description of the studies

**Fig 1:**
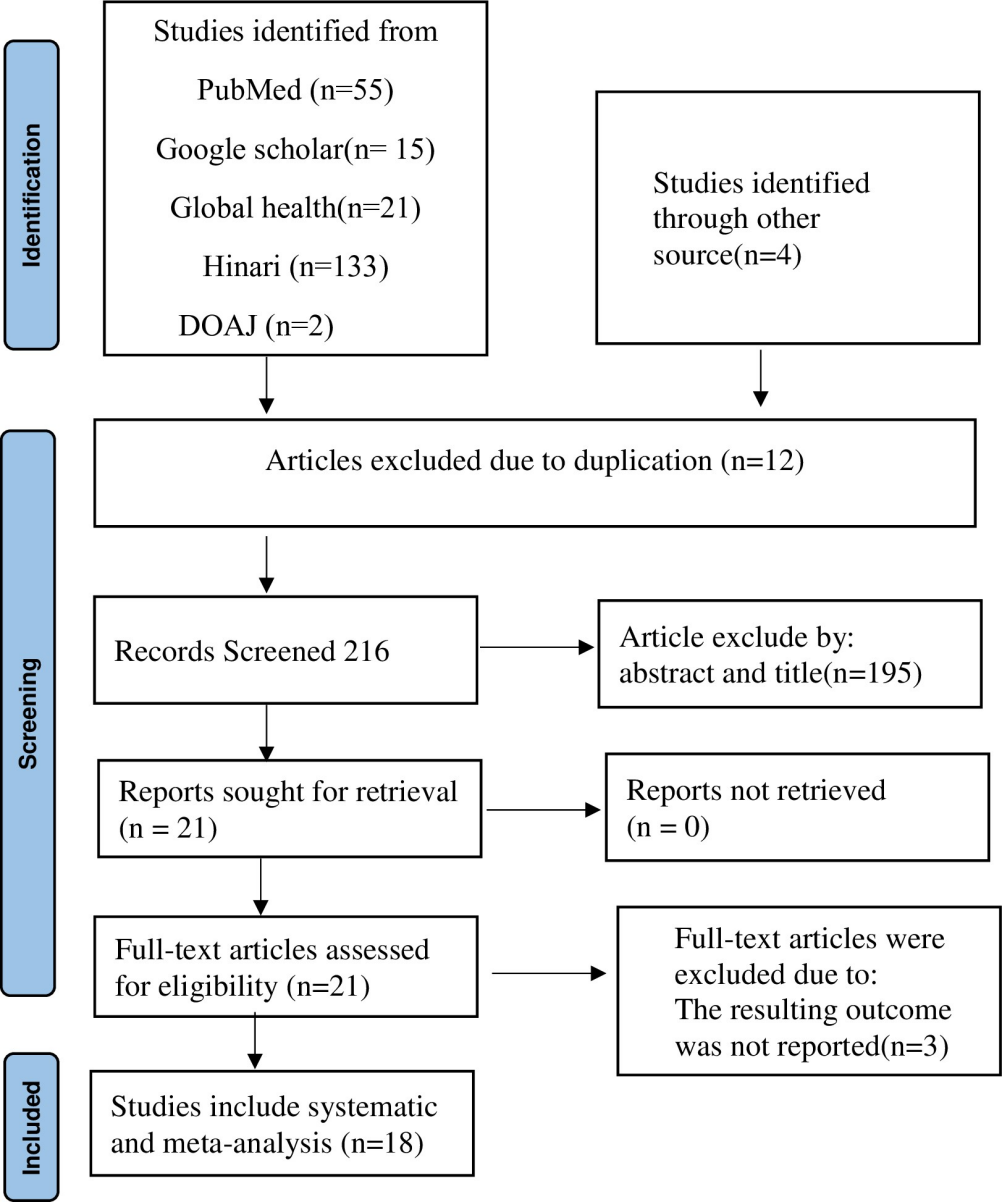
PRISMA flow diagram of the study selection process for systematic review and meta-analysis.

In our literature, a total of 228 articles from various sources were found using Pub Med, Google Scholar, Global Health, and the Hinari database. Of these, 12 duplicate articles were removed using ENDNOTES and manual tracing. After duplicates were removed, 216 studies remained, and 195 articles were removed based on an assessment of their abstracts and titles. A full-text review was performed for 21 articles and 3 articles were excluded due to the result outcome was not reported. The remaining 18 articles that met the eligibility criteria were included in this literature review (Fig 1).

### Characteristics of the included studies

A total of 18 studies with 519,327 participants were included for systematic review and meta-analysis, including 10,302 cases of congenital anomalies. All included studies that used only cross-sectional studies [16–27] were used to estimate the pooled prevalence of congenital anomalies in Ethiopia. Case-control studies [28–33] and cross-sectional were used to estimate the pooled prevalence of associated factors of congenital anomalies. The year of publication of the included studies ranges from 2015 to 2023.

### Prevalence of congenital anomalies

**Fig 2:**
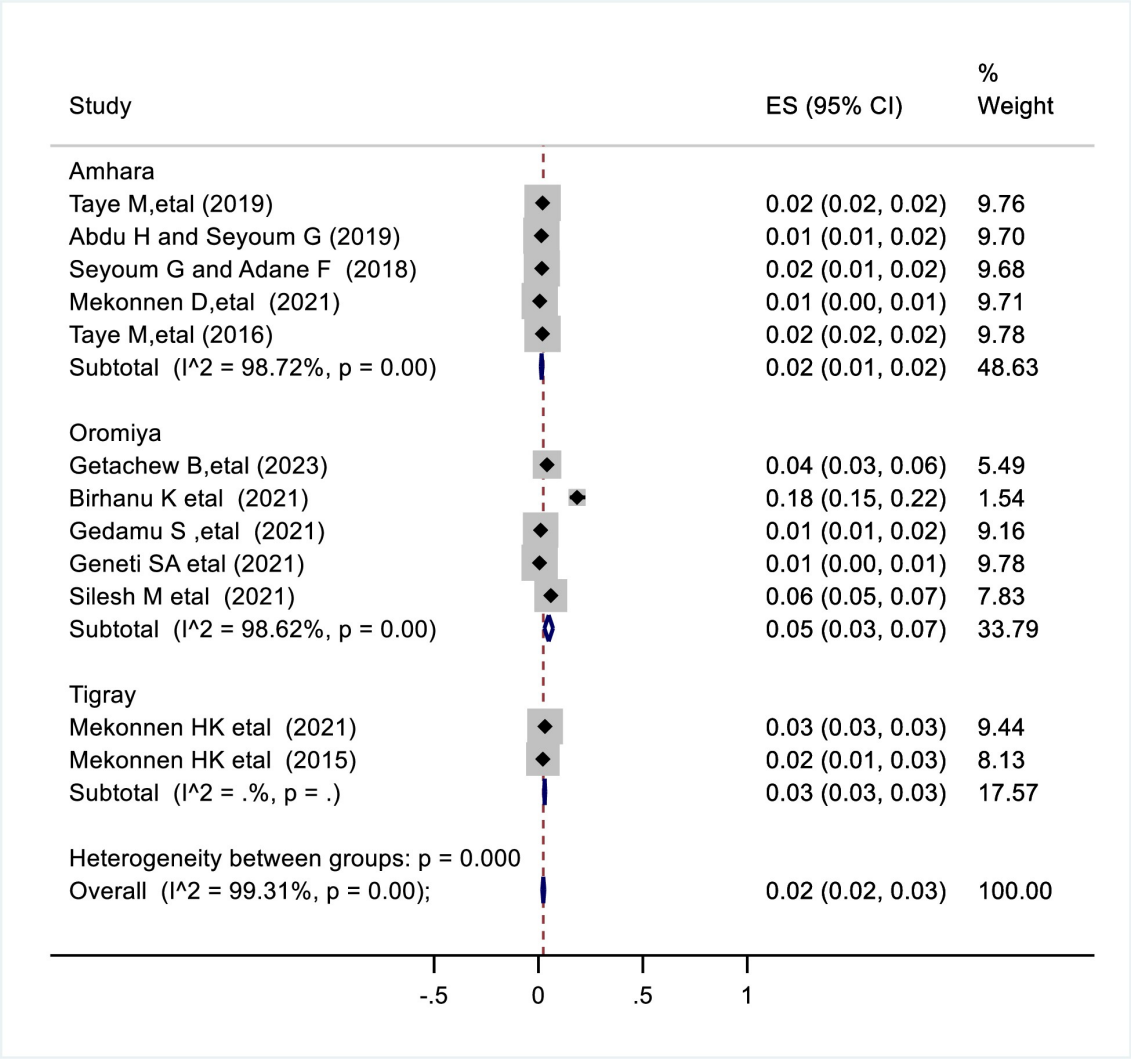
Forest plot shows pooled prevalence of congenital anomalies in Ethiopia.

The overall estimate of congenital anomalies from the studies in Ethiopia was 2% (95% CI: 0.02, 0.03%). When a random effects model was used in this meta-analysis, a high level of heterogeneity between studies was observed, as shown by the I^2^ statistic (I^2^ = 99.31%, p = 0.001) (Fig 2). The prevalence of congenital anomalies was compared across studies based on various participant variables using subgroup analysis by region in Ethiopia. This study found that the Amhara region had the lowest prevalence of congenital anomalies at 2% (95% CI: 0.01, 0.02) and the highest prevalence at 5% (95% CI: 0.03, 0 .07) was recorded in the region Oromia (Fig 2**).**

### Types of congenital anomalies

The total number of cases was used to estimate all forms of congenital anomalies. According to Table 2, the combined prevalence of neurological defects was 48% (95% CI: 0.40, 0.57). The estimated pooled prevalence of orofacial clefts was 19% (95% CI: 0.12, 0.26) (Table 2), followed by the musculoskeletal defect 12% (95% CI: 0.07, 0.17) (S 1 Fig). According to Table 2, the pooled prevalence of GIT was 13% (95% CI: 0.09, 0.18). Defect of the cardiovascular system:7% (95% CI: 0.04, 0.11) and Down syndrome: 5% (95% CI: 0.02, 0.07as indicated in Table 2.

**Table 2:** Pooled prevalence of different types of birth defect in Ethiopia, 2023.

Types of birth defect	Pooled prevalence	95% CI
Musculoskeletal system defect	12	**(**0.07–0.17)
Cardiovascular system defects	7	(0.04–0.11)
Gastrointestinal system defect	13	(0.09–0.18)
Orofacial clefts	19	(0.12–0.26)
Down syndrome	5	(0.02–0.07)
Neural tube defect	48	(0.40–0.57)

### Risk factors of congenital anomalies in Ethiopia

**Fig 3:**
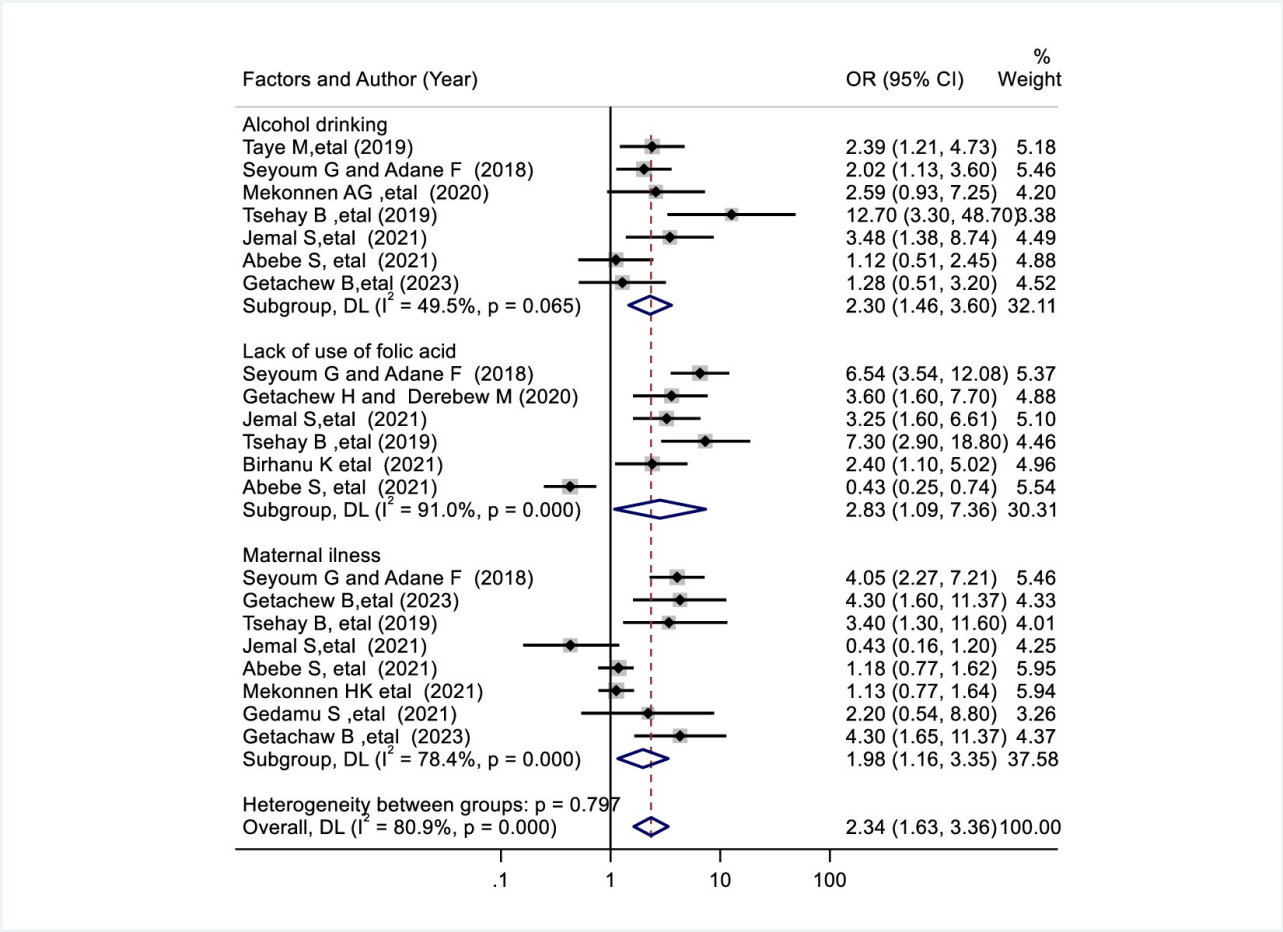
Forest plot shows factors associated with congenital anomalies in Ethiopia.

Thirteen of the 18 primary studies on congenital malformations examined factors associated with birth defects and assessed them using random effects models. For description purposes, the graph was divided into three figures. Alcohol consumption during pregnancy and congenital anomalies were examined using data from six studies [17,18,28–31,33]. The result showed that alcohol consumption during pregnancy was significantly associated with congenital anomalies. Congenital anomalies were 2.28 times more likely to occur in mothers who consumed alcohol than in mothers who did not consume alcohol (pooled odds ratio: 2.28, 95% CI: 1.54, 3.38) (Fig 3). Six primary studies were used to examine the association between not use of folic acid supplements and congenital anomalies [17,19,29,30,32,33]. According to the results, congenital anomalies were 2.83 times more common in mothers who did not take folic acid than in mothers who took folic acid (pooled odds ratio: 2.83, 95% CI: 1.09, 7.36) (Fig 3). Using seven studies [17,18,20,21,29,30,33] the relationship between maternal illness and congenital anomalies was studied. The result suggested that maternal illness during pregnancy was significantly associated with congenital anomalies. Congenital anomalies were 1.79 times more common in sick mothers than in healthy mothers (pooled odds ratio: 1.79, 95% CI: 1.03, 3.10) (Fig 3).

**Fig 4:**
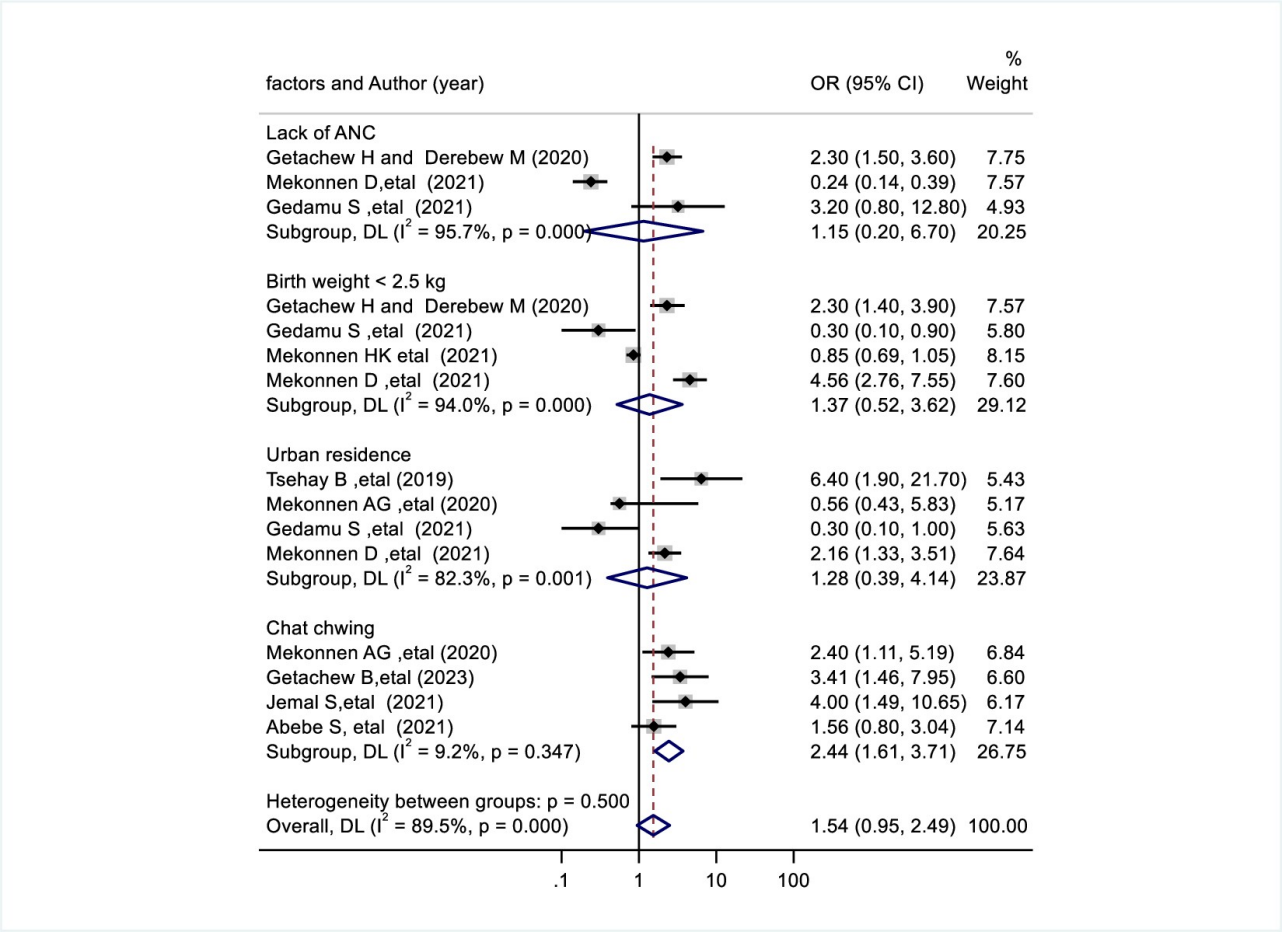
Forest plot shows factors associated with congenital anomalies in Ethiopia.

The association between khat (*Catha edulis*) chewing during pregnancy and congenital anomalies was evaluated by four studies [18,30,31,33]. Congenital anomalies were found 2.44 times more common in mothers who consumed khat (*Catha edulis*) than in mothers who did not consume khat (pooled odds ratio: 2.44, 95% CI: 1.61, 3.71) (Fig 4). Lack of ANC was evaluated using three studies [21,22,32] (pooled odds ratio 1.15,95% CI:0.20,6.70), Four articles reported birth weight <2.5kg [20–22,32] (pooled odds ratio: 1.37, 95% CI: 0.52, 3.62), and four studies reported urban residence [21,22,29,31] (pooled odds ratio: 1.28, 95%CI: 0.39, 4.14) during pregnancy there was no significant association with congenital anomalies (Fig 4).

**Fig 5:**
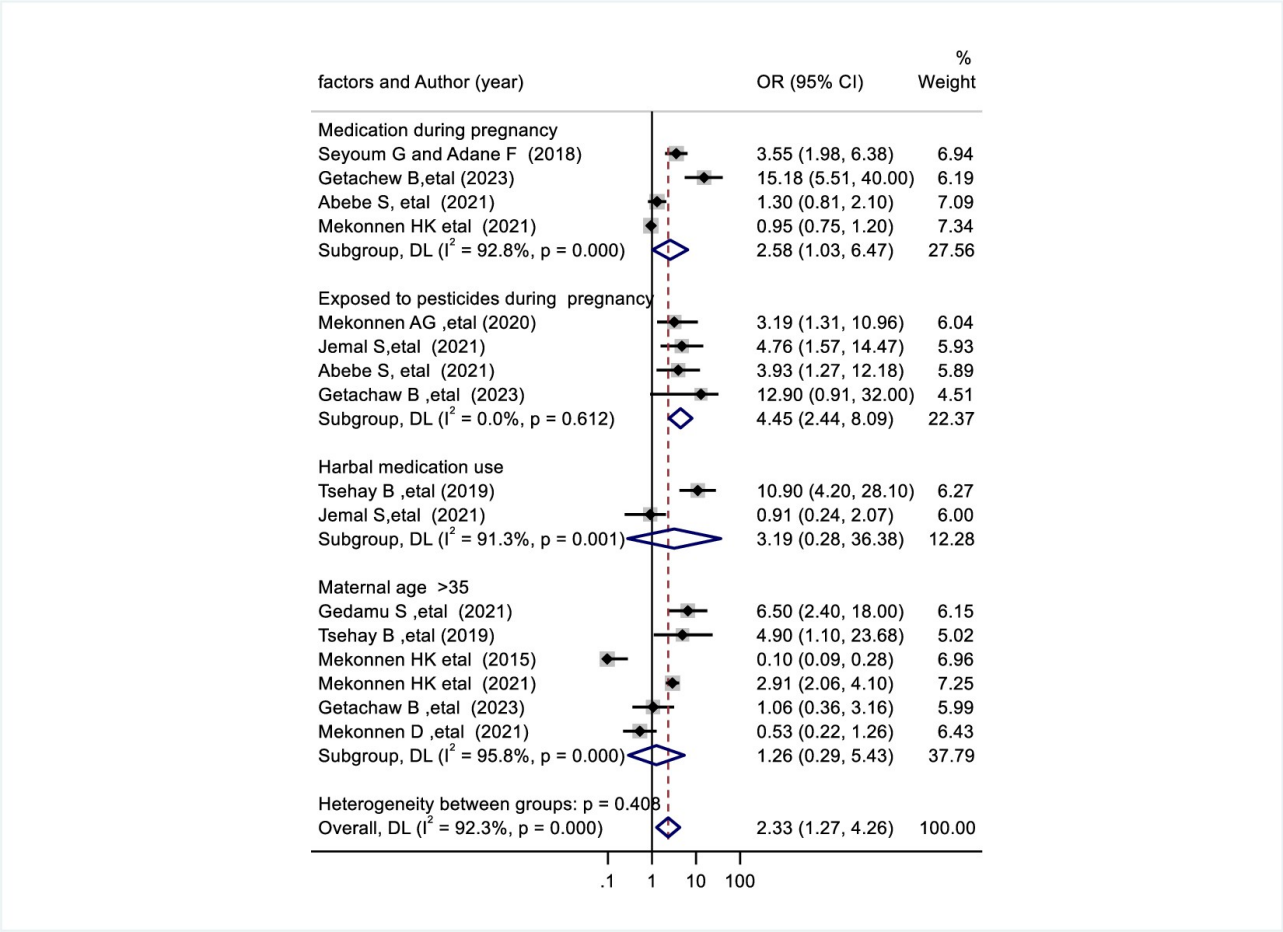
Forest plot shows factors associated with congenital anomalies in Ethiopia.

The effect of exposure to teratogenic medication during pregnancy was evaluated using four studies [17,18,20,33]. In this study, exposure to teratogenic medication during pregnancy was found to be significantly associated with congenital anomalies. Congenital anomalies were found 2.58 times more often in mothers who took teratogenic drug**s** than in mothers who did not the take drug (pooled odds ratio: 2.58, 95%CI: 1.03, 6.47) (Fig 5). The association between exposure to pesticides during pregnancy and congenital anomalies was evaluated using four studies [18,30,31,33]. Congenital anomalies were 4.45 times more common in mothers exposed to pesticides than in mothers not exposed to pesticides (pooled odds ratio: 4.45, 95%CI: 2.44, 8.09) (Fig 5). Herbal medications during pregnancy were evaluated in two studies [29,30] (pooled odds ratio: 3.19, 95%CI: 0.28, 36.38), and maternal age > 35 years was evaluated in six studies [18,20–23,29] (pooled odds ratio: 1.26, 95% CI:0.29, 5.43) were both no significant associated with congenital anomalies (Fig 5).

### Meta-regression analysis

Meta-regression analysis was conducted to specify the cause of heterogeneity. According to our systematic review and meta-analysis sample size (p = 0.001) and year of publication (p = 0.001) were significant for the source of heterogeneity.

### Publication bias

**Fig 6:**
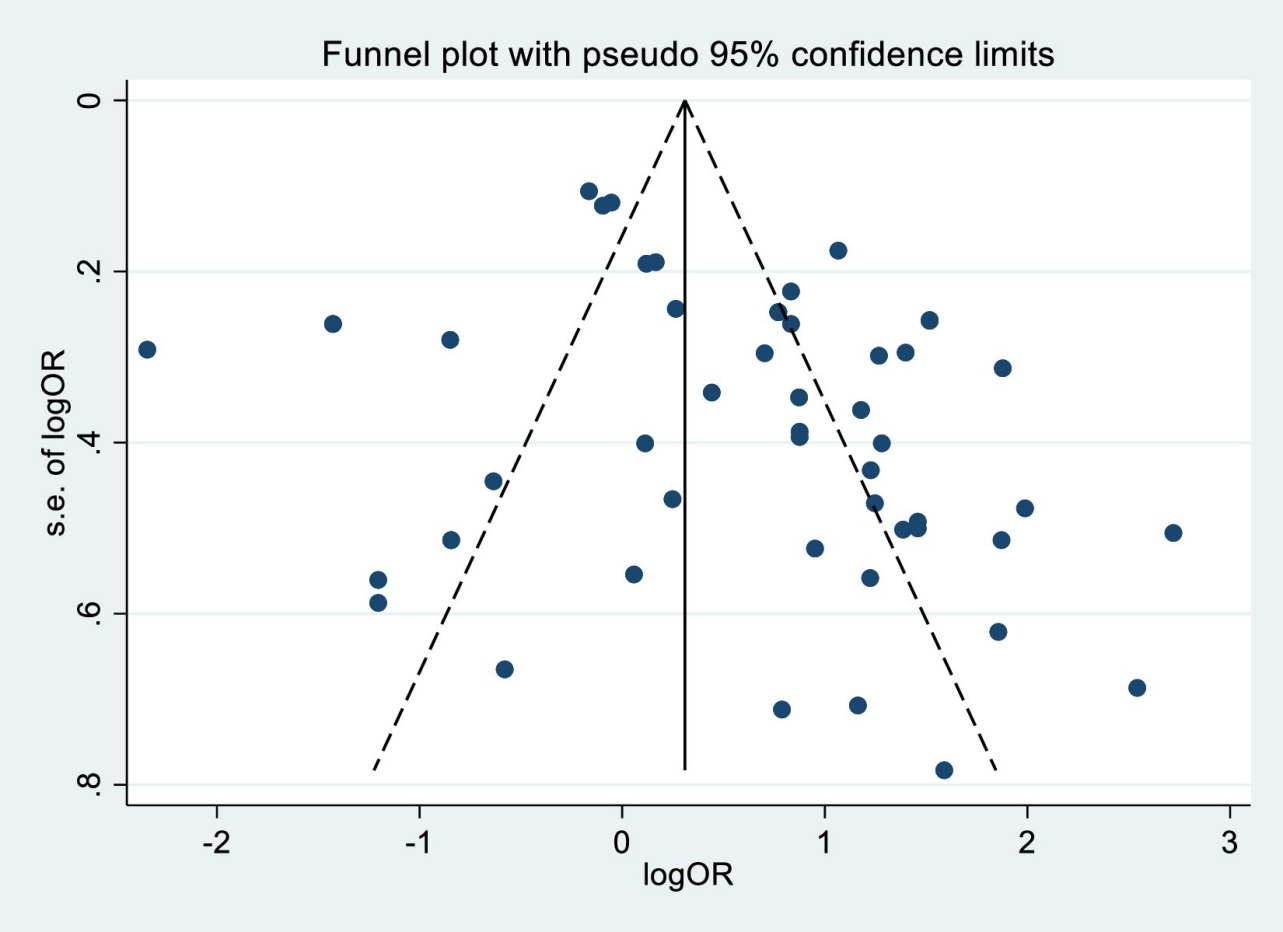
Funnel plot illustrating publication bias of included studies for factors of congenital anomalies in Ethiopia.

The funnel plot for Fig 6 shows this visual evidence of standard error publication bias with a log of associated factors of congenital anomalies. The funnel plot was approximately asymmetrical, suggesting publication bias. The Egger test was used as a statistical method to assess publication bias; the effects of research on the H0 test are significant (Egger test, p = 0.006). Therefore, the trim and fill analysis method was used to adjust for publication bias.

## Discussion

Congenital anomalies are a significant cause of death and disability worldwide. The World Health Organization reports that approximately 6% of children are born with such anomalies, resulting in the deaths of 295,000 infants annually across the globe. This systematic review and meta-analysis is the first of its kind in Ethiopia, as no other report has documented the pooled prevalence of congenital anomalies in the country. The results of this review show a pooled prevalence of 2% (95% CI: 0.02, 0.03) in Ethiopia, which means that 20 infants out of every 1000 births in the country are born with congenital anomalies. Our findings also reveal that the Oromia region is the most affected, compared to the Amhara and Tigray regions. The pooled prevalence of congenital anomalies in Ethiopia is higher than that reported in studies conducted in Kenya, where 6.3 congenital anomalies are reported for every 1000 births [36], and in Britain, where the number is 12.9 per 1000 births [37]. However, the pooled prevalence of congenital anomalies in Ethiopia is comparable to that reported in a review conducted in Iran [38]. The prevalence of congenital anomalies in Ethiopia was marginally lower than the prevalence in Central, Southern, Eastern, and Western African countries. The variance in rates may be due to environmental, racial, sample size, and attention given to birth defect registration [7]. Our findings indicate that neural tube defects were the most frequent type of congenital anomaly, followed by orofacial cleft defects. Other congenital anomalies, such as gastrointestinal defects, musculoskeletal defect, cardiovascular defects, and Down syndrome were identified as less frequent congenital anomalies. This discovery is consistent with the results of a study conducted in Guatemala [39]. A systematic review conducted in various African countries also indicates that neural tube defects are common in Ethiopia. The high occurrence of neural tube defects can be attributed to a lack of antenatal care follow-up and folic acid supplementation during pregnancy [40].

The occurrence of congenital anomalies is influenced by various factors. In our meta-analysis, we examined that maternal alcohol consumption, maternal illness, exposure to pesticides, khat (*Catha edulis*) chewing, lack of folic acid supplementation, and medication during pregnancy, showed a statistically significant association with congenital anomalies (p<0.05). Our results indicated that Congenital anomalies were 2.28 times more likely to occur in mothers who consumed alcohol than in mothers who did not consume alcohol. This result is lined with a systematic review done in sub-Saharan African countries and it is also supported by a cohort study done in Europe [7,41]. Our findings revealed that congenital anomalies were 2.83 times more common in mothers who did not take folic acid than in mothers who took folic acid during pregnancy. This result is consistent with a systematic review conducted in sub-Saharan African countries [7]. It is also supported by studies carried out in Pakistan, Uganda, Italy, and the Netherlands [42–44], which reported that folic acid supplementation during pregnancy can decrease the likelihood of having malformed infants. This may be because folic acid supplementation is essential for the synthesis of various important biomolecules, such as proteins, lipids, and nucleic acids, which are critical for cell growth [45,46]. Folic acid acts as a co-enzyme in different biochemical pathways, including the synthesis of DNA, RNA, and amino acids. Therefore, during pregnancy, greater amounts of folic acid are required due to the rapid rate of cell and tissue growth [46].

Maternal illness during pregnancy has been found to increase the propensity for congenital anomalies by a factor of 1.79, compared to healthy mothers. This outcome is consistent with other systematic reviews, which suggest that maternal illness is associated with a higher likelihood of having a child with congenital malformations, as compared to non-ill women [47]. Additionally, a large population-based case-control study conducted in Atlanta [48] supports this finding. The direct correlation between maternal illness and congenital anomalies may be attributed to the use of various drugs to treat illness during pregnancy. Based on our findings, chewing khat (*Catha edulis*) during pregnancy increases the likelihood of congenital anomalies by 2.44 times compared to those who do not chew khat. This aligns with a study conducted in the Bale zone, which revealed that women who chewed khat (*Catha edulis*) during the preconception period were three times more likely to have malformed infants than non-chewers [49]. This could be attributed to the presence of cathinone and cathine chemicals in khat (*Catha edulis*), which can cross the placental barrier and lead to fetal growth retardation by reducing blood flow from the mother to the fetus [5].

Our meta-analysis revealed that congenital anomalies were found 2.58 times more often in mothers who took teratogenic drugs during pregnancy than in mothers who did not take drugs. Although it opposes the result of a hospital-based study in Nigeria [50], our findings align with other population-based studies, which indicate that taking antiepileptic drugs during pregnancy is twice as risky for congenital anomalies [51]. Our results are also supported by another study that revealed taking antihypertensive drugs as a causal factor of congenital anomalies [52]. This association may be due to some drugs that can transfer to the fetus through the placenta and may alter the normal physiology of the infant [53]. The findings of our review suggest that during pregnancy, maternal exposure to pesticides could lead to a 4.45 times higher chance of infant congenital anomalies in comparison to mothers who did not have any exposure to chemicals. Additionally, Garcia has reported that exposure to chemical pesticides can result in defects of the central nervous system, oro-facial, and musculoskeletal systems [54]. This is further supported by a community-based study in northern Italy, which indicated that exposure to benzene and PM10 is linked to musculoskeletal and chromosomal abnormalities [55]. Although the action mechanism of pesticides on infants is not well understood, it is possible that genotoxicity and mutation could be enhanced by organophosphates present in pesticides [56].

## Conclusion

Congenital anomalies constitute a significant public health challenge of global proportions, particularly in developing nations such as Ethiopia. Our review indicates that the prevalence of congenital anomalies is higher in Ethiopia than in the United Kingdom and Kenya. The most common type of congenital anomaly in Ethiopia was neural tube defects, followed by oro-facial cleft. Cardiovascular defects and Down syndrome were less prevalent in Ethiopia. Factors such as alcohol consumption, inadequate intake of folic acid, khat (*Catha edulis*) chewing, maternal diseases, exposure to pesticides, and use of medication during pregnancy were identified as potential contributors to congenital abnormalities in Ethiopia.

## Strength and limitation

The present systematic review and meta-analysis stands out for being the pioneer study to tackle the pooled prevalence of congenital anomalies in Ethiopia. Multiple databases were employed in the quest for related articles. However, we must acknowledge that this review is not without limitations, given that the pooled estimate does not account for home deliveries. Furthermore, it is worth noting that all studies encompassed in this review were institutional-based and limited on articles written in English language. Moreover, the variability in sample size among these studies could potentially impact the combined estimate of congenital anomalies.

## Supporting information

S1 Checklist PRISMA 2009 check list used for prevalence and associated factors affecting congenital anomalies in Ethiopia: Systematic review and meta-analysis. (PDF)

S1 Fig The Forest plot shows pooled prevalence of musculoskeletal defects among total congenital anomalies in Ethiopia. (TIF)

S2 Fig The forest plot shows pooled prevalence of cardiovascular system defects among total congenital anomalies in Ethiopia. (TIF)

S3 Fig The forest plot shows pooled prevalence of gastrointestinal system defects among total congenital anomalies in Ethiopia. (TIF)

S4 Fig The forest plot shows pooled prevalence of orofacial defect among total congenital anomalies in Ethiopia. (TIF)

S5 Fig The forest plot shows pooled prevalence of Down syndrome among total congenital anomalies in Ethiopia. (TIF)

S6 Fig The forest plot shows pooled prevalence of neural tube defects among total congenital anomalies in Ethiopia. (TIF)

## Abbreviations

JBI: Joanna Briggs Institute
OR: Odds Ratio
PRISMA: Preferred Reporting Items for Systematic review and Meta-analysis
WHO: World Health Organization
GIT: gastrointestinal tract
