# Supplementary figures and images for: Prevalence and associated factors of congenital anomalies in Ethiopia: A systematic review and meta-analysis
Source: PLoS One. 2024 Apr 30;19(4):e0302393. doi: 10.1371/journal.pone.0302393 (PMC11060542; doi:10.1371/journal.pone.0302393)

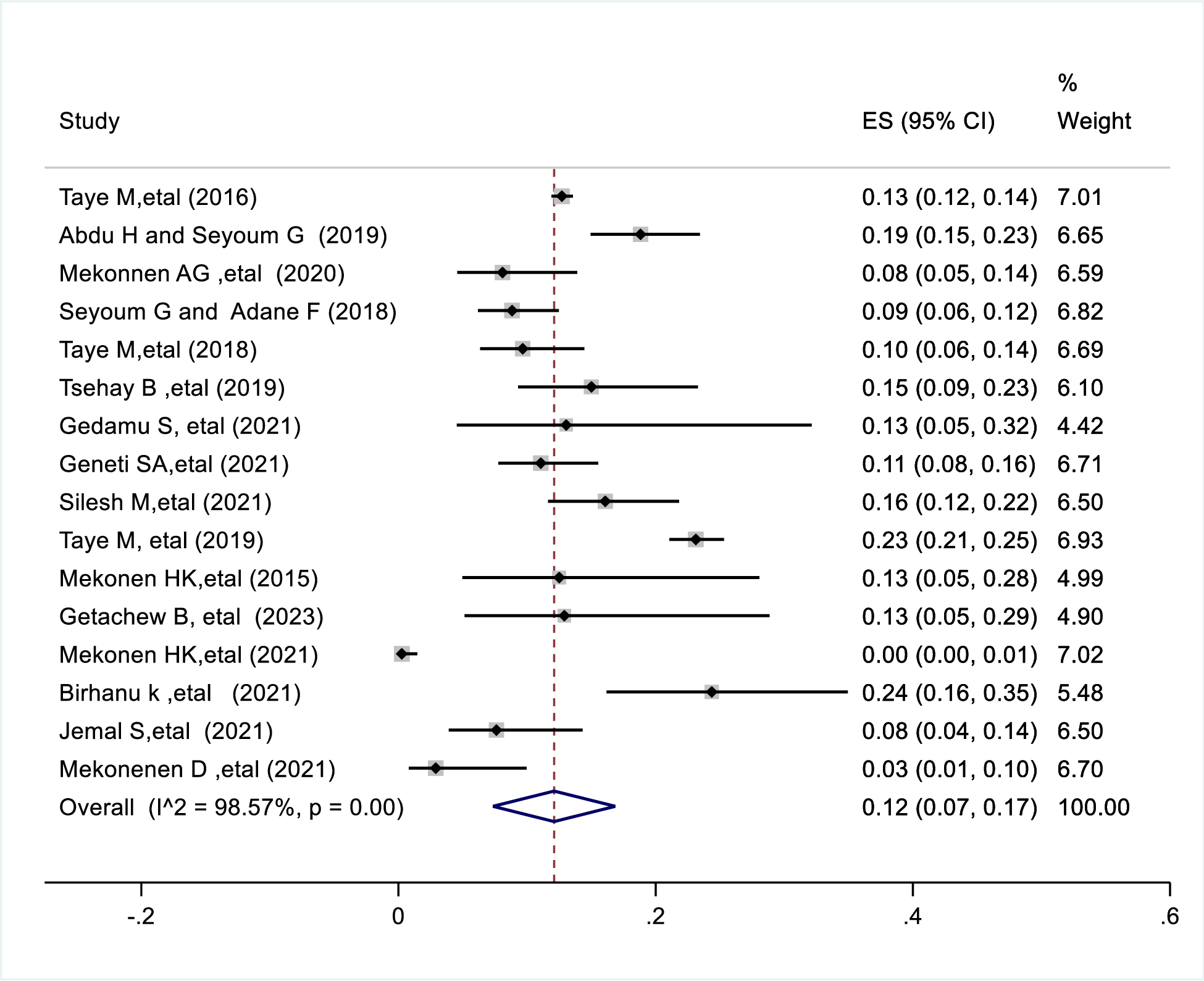

Supplement: S1 Fig — (TIF) [file pone.0302393.s002.tif]

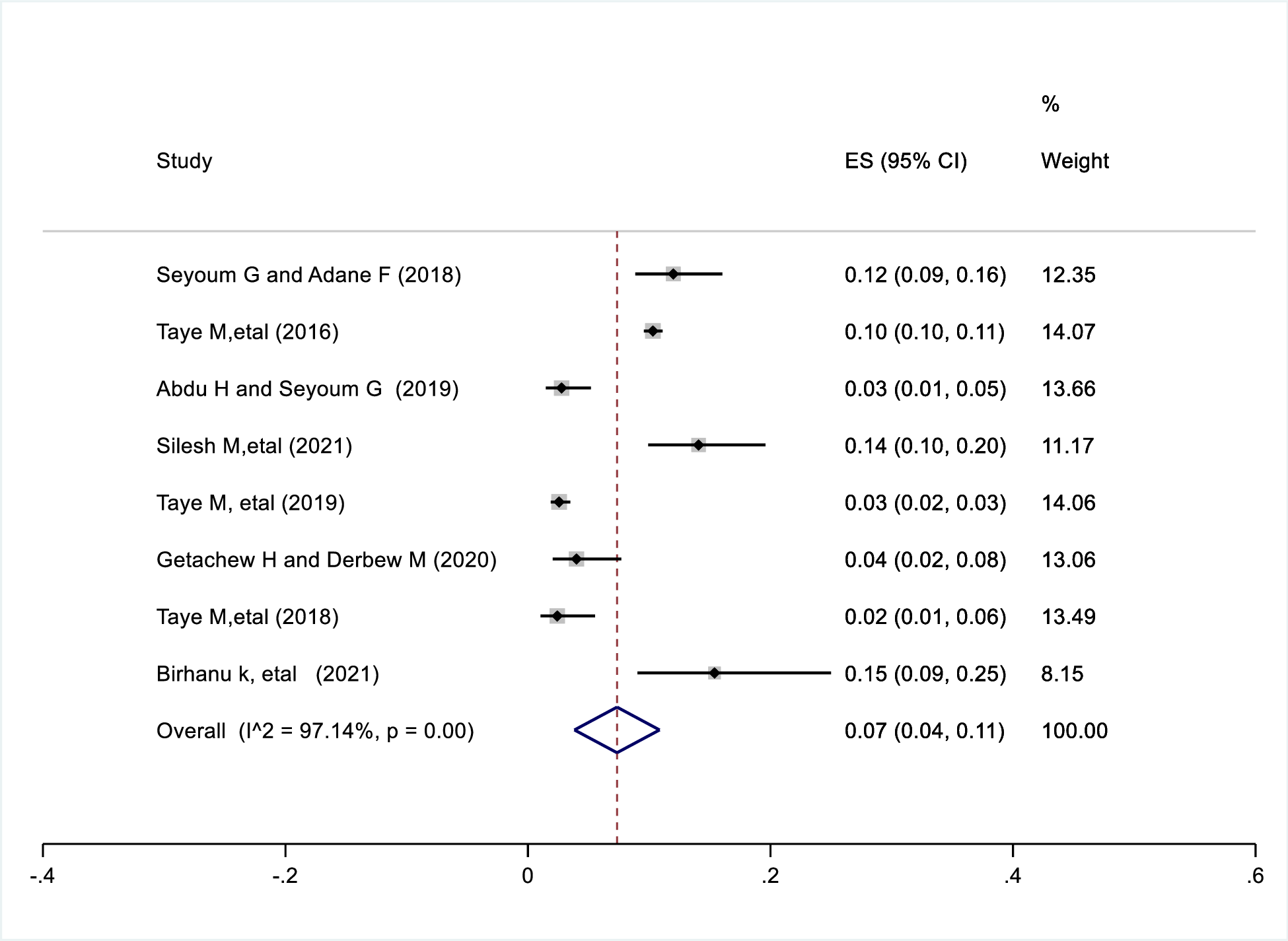

Supplement: S2 Fig — (TIF) [file pone.0302393.s003.tif]

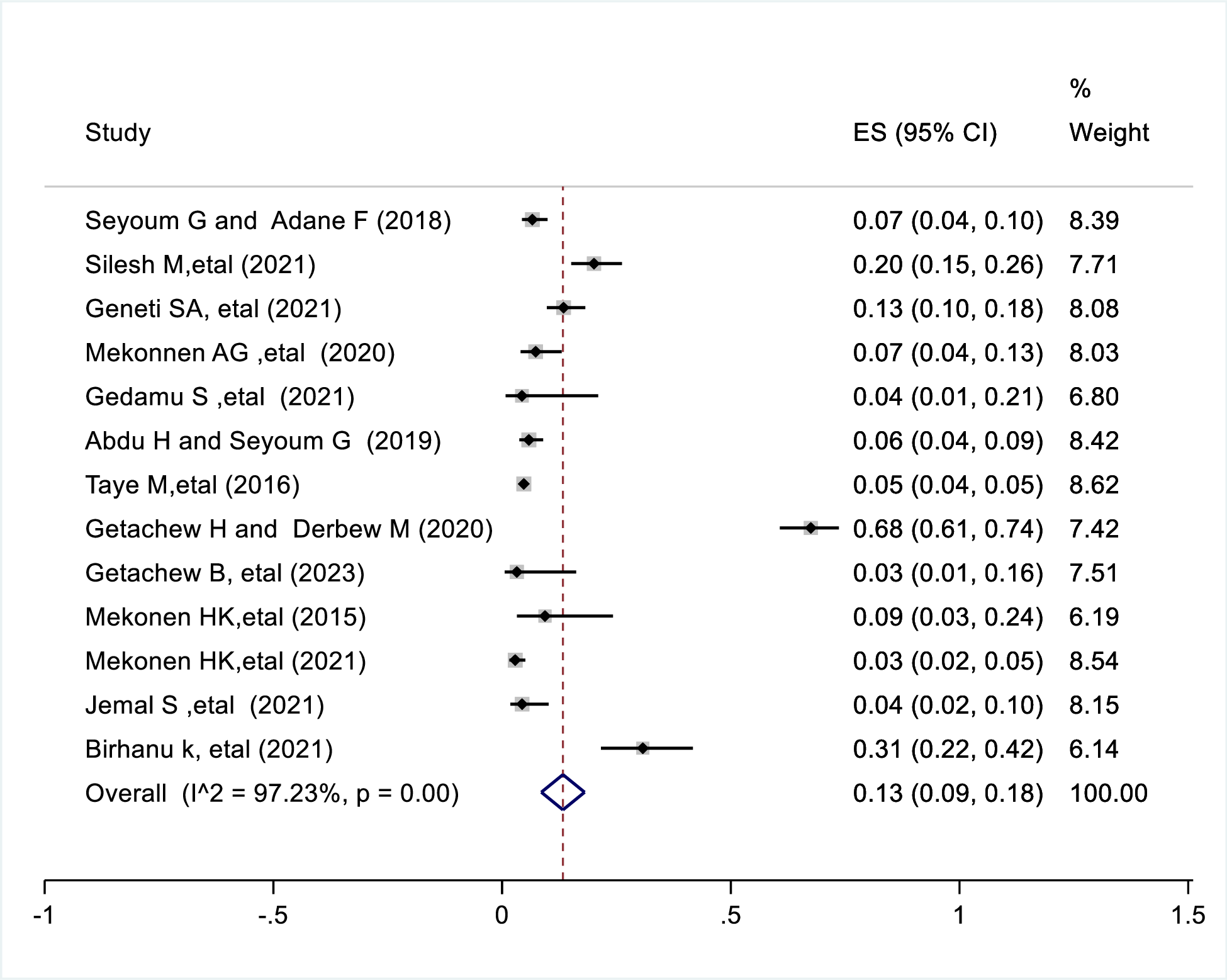

Supplement: S3 Fig — (TIF) [file pone.0302393.s004.tif]

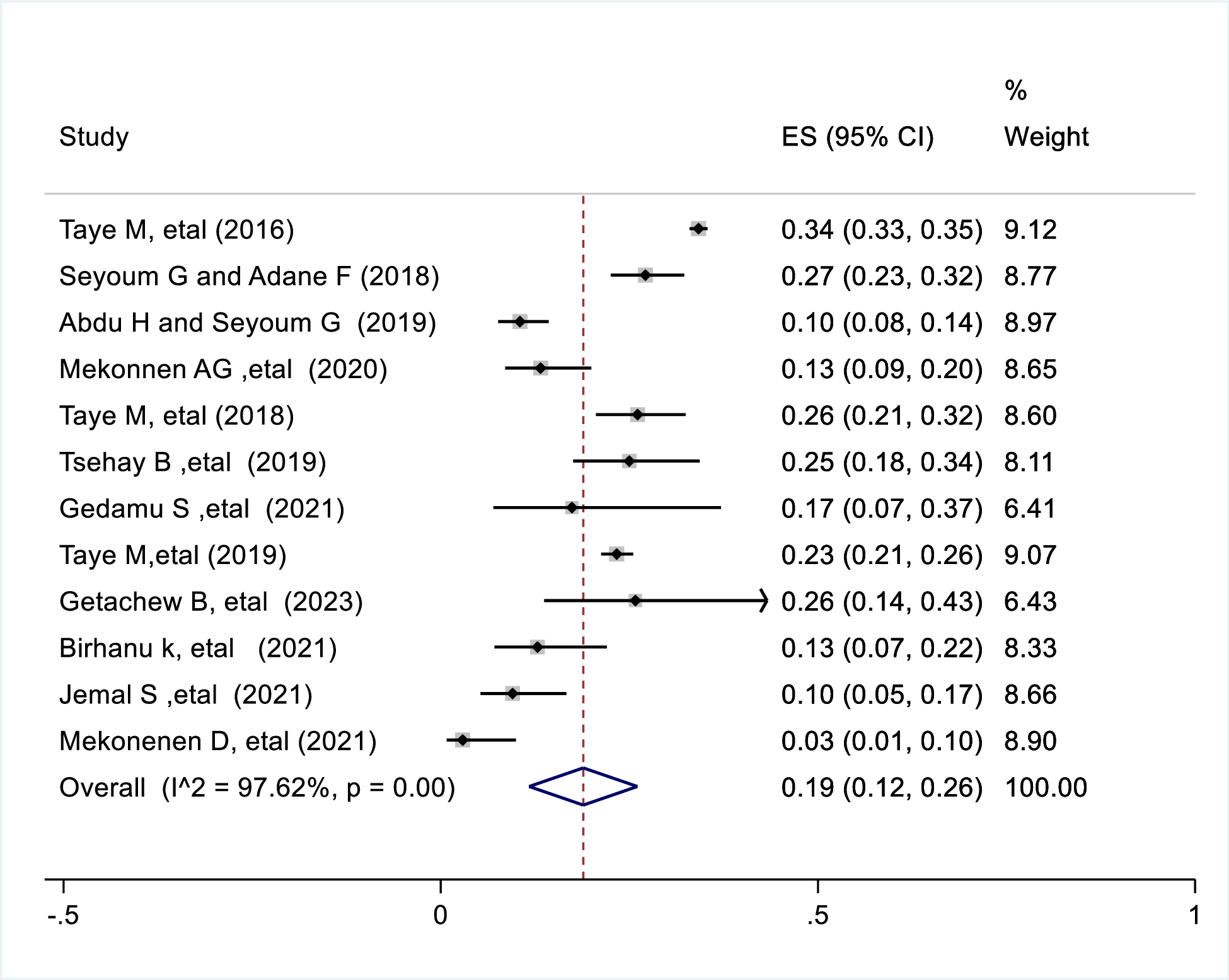

Supplement: S4 Fig — (TIF) [file pone.0302393.s005.tif]

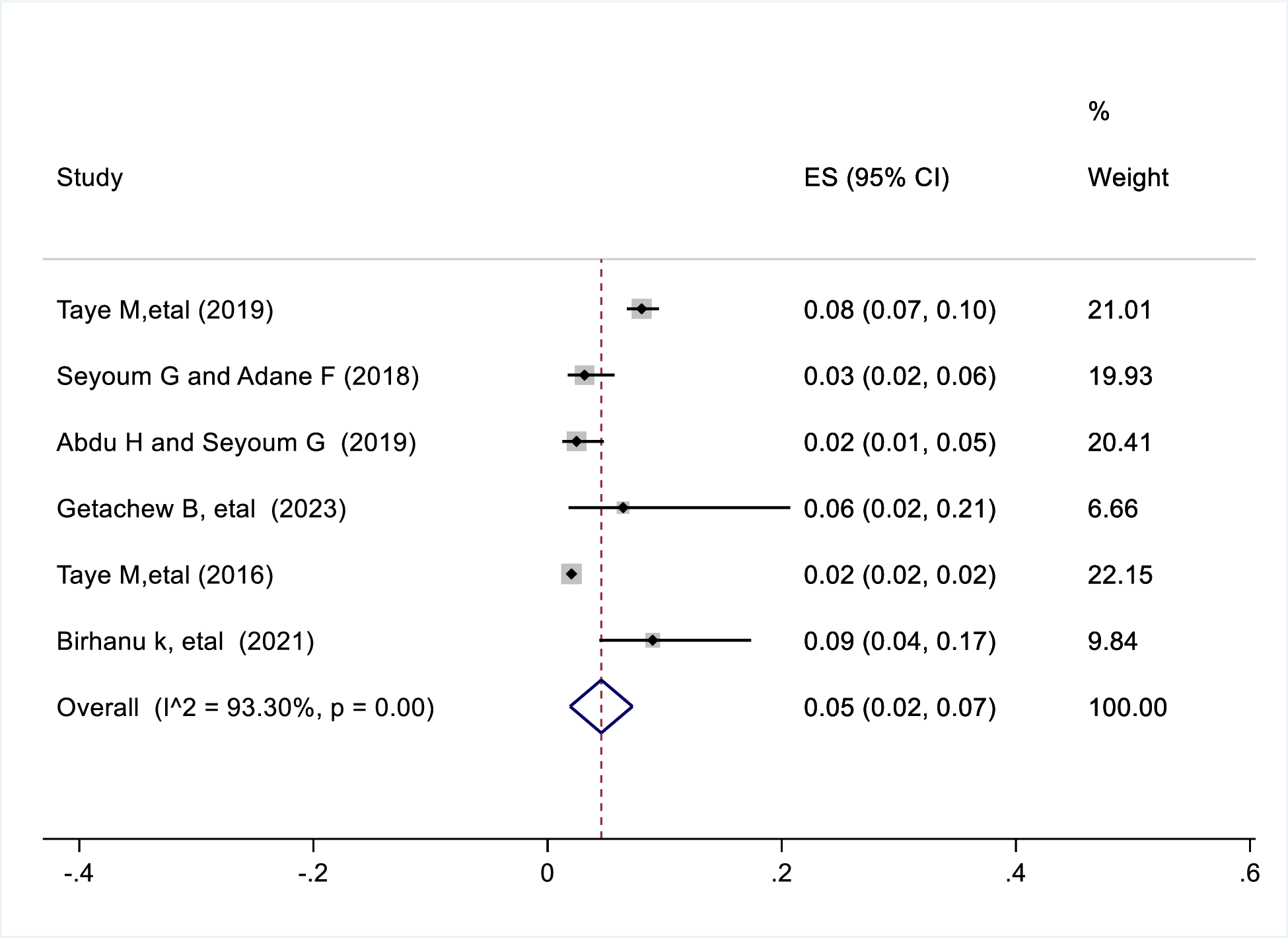

Supplement: S5 Fig — (TIF) [file pone.0302393.s006.tif]

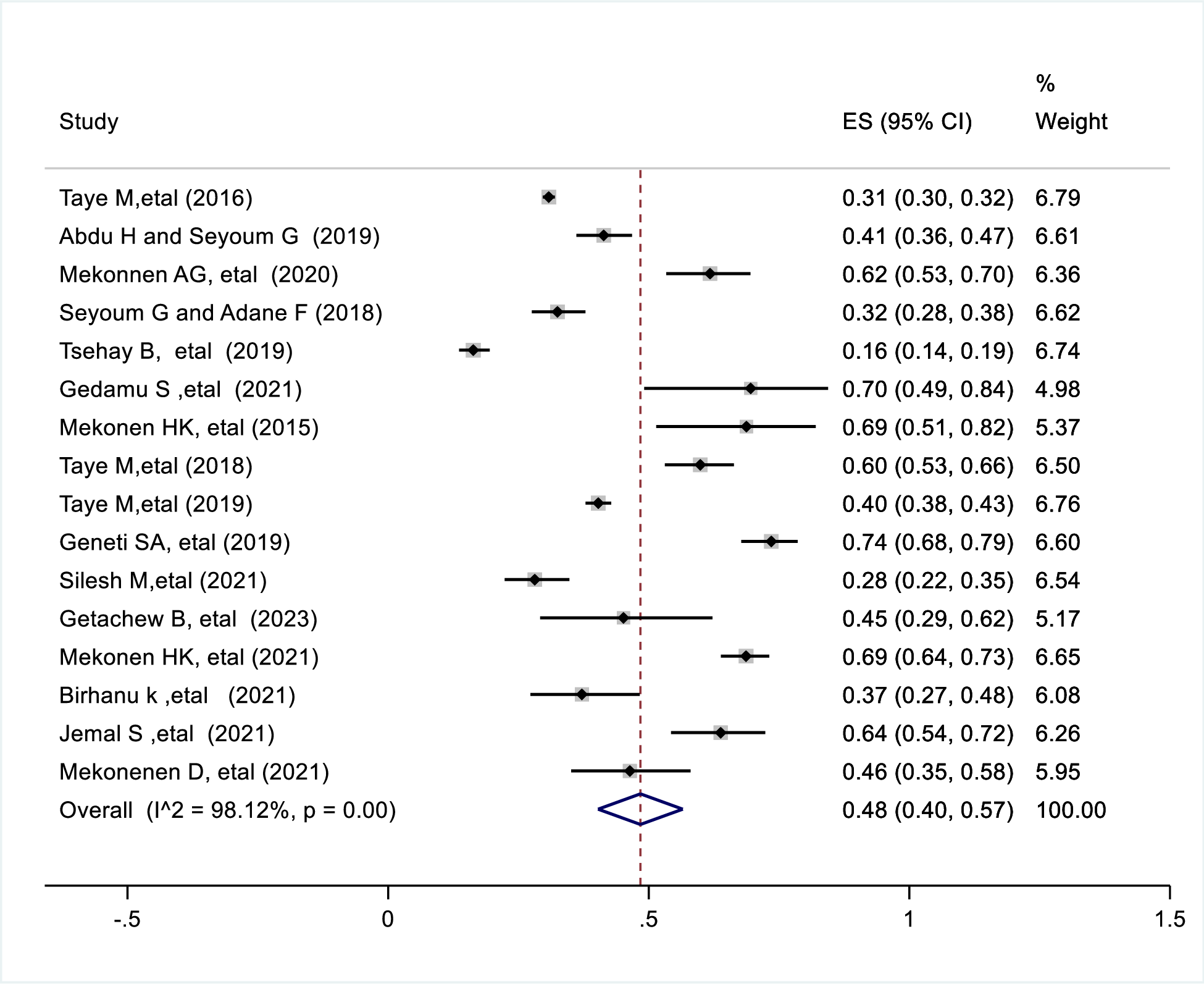

Supplement: S6 Fig — (TIF) [file pone.0302393.s007.tif]
